# Post-refinement method for snapshot serial crystallography

**DOI:** 10.1098/rstb.2013.0330

**Published:** 2014-07-17

**Authors:** Thomas A. White

**Affiliations:** Center for Free-Electron Laser Science, Deutches Elektronen-Synchrotron DESY, Notkestrasse 85, 22607 Hamburg, Germany

**Keywords:** data analysis, post-refinement, scaling, serial crystallography

## Abstract

A post-refinement procedure has been devised for ‘snapshot’ diffraction data consisting entirely of partially recorded reflections, each diffraction pattern from a crystal in an orientation unrelated to the others. Initial estimates of the diffraction geometry are used to calculate initial partialities, which are then used to scale the entire dataset together to produce initial estimates of the fully integrated intensities. The geometrical parameters for each pattern are then refined to maximize the agreement between these estimates and the calculated intensities in each pattern, and the procedure repeated iteratively. The performance of the procedure was investigated using simulated data and found to yield a significant improvement in the data quality.

## 1. Introduction

In ‘serial femtosecond crystallography’ (SFX), a single X-ray diffraction pattern is acquired from each one of a very large number of crystals, delivered into the path of an X-ray free-electron laser (FEL) beam by a liquid injection technique which results in all the crystals having random and unrelated orientations [1]. Because a single pulse of FEL radiation destroys each crystal, controlled rotation or oscillation of the crystals is impossible, and there is no possibility of returning to a particular crystal to acquire more data. It is the very short duration of the X-ray pulse, only a few tens of femtoseconds, which allows a diffraction pattern to be recorded despite the destruction of the crystal [2]. Owing to the resulting lack of rotation or oscillation, and the use of an X-ray beam with small bandwidth and convergence angle (although generally neither monochromatic nor perfectly collimated), it can be expected that the reflections in each diffraction pattern would be partially recorded. In the experiments of this kind performed to date, integrated intensities have been calculated using the so-called Monte Carlo technique in which the full reflection intensities are determined simply by taking the mean of sufficiently large number of measurements [3], and it has been demonstrated several times that this method can indeed produce useful intensities from experimental data [4].

The Monte Carlo method makes no attempt to assign partialities to any of the reflections. Assuming that the unknown partialities of the reflections really are the dominant error source which necessitates such a large volume of data in SFX experiments, a great improvement in the final data quality could be achieved by assigning values to them. However, useful partiality estimates cannot usually be made with the initial geometrical parameters (unit cell parameters and crystal orientation) given by the indexing procedure. ‘Post-refinement’ is the method by which these parameters can be refined in order to get the best agreement between the ‘scaled up’ partial intensity and some reference intensity [5], improving the accuracy of the geometrical parameters to a point where useful partiality estimates can be made. The need for special data processing techniques for the ‘one crystal–one photograph’ situation was identified as early as 1979, when Winker *et al.* [6] reported a suitable post-refinement procedure. However, their method relies on having fully recorded ‘reference’ measurements for at least a fraction of the partial reflections, whereas in the SFX situation, there are no fully recorded reflections whatsoever.

Rossmann & van Beek [5] identified two methods for handling partially recorded reflections. The first ‘method of summed partials’ involves summing partial reflections from adjacent images in a rotation series and is obviously not applicable here. The second ‘method of scaled partials’ involves taking an average of individual estimates of the fully integrated intensity after individually correcting each partial intensity using its calculated partiality. A suitable scaling method for SFX could consist of incorporating post-refinement in this method and iterating, performing post-refinement of each diffraction pattern against the estimates of the full intensities at each step, calculating new overall scaling factors and then making improved estimates of the full intensities before repeating. This article describes an implementation of this ‘method of scaled partials with post-refinement’, a processing technique with appears not to have been described so far. Using simulated test data, it will be demonstrated that post-refinement can be applied to a dataset which consists entirely of partially recorded reflections, yielding sufficiently accurate partiality estimates to allow significant improvements to be made to the quality of the final intensity measurements.

## 2. Geometrical model for partiality

For this work, the diffraction geometry was modelled as described previously with the partiality of a reflection being defined as the volume fraction of a sphere, centred on the reciprocal lattice point, which is within the region of reciprocal space covered by the nest of Ewald spheres representing the different X-ray wavelengths (to model spectral bandwidth) and incident angles (to model beam convergence) [7]. The model is similar to that described by Rossmann *et al.* [8].

**Figure 1.**
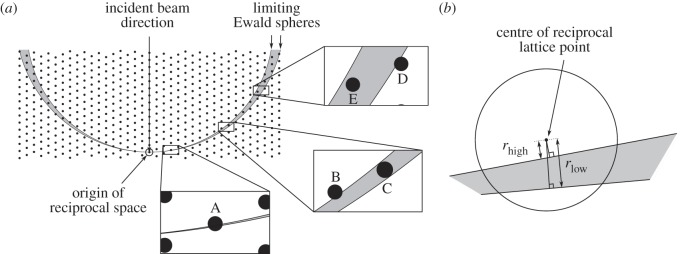
(*a*) Cross section of reciprocal space showing the volume between the limiting Ewald spheres within which reflections can be excited (shaded in grey). The enlarged regions show five reflections, labelled A–E, which have different values of partiality and Lorentz factor. (*b*) Further enlargement of reflection ‘A’ showing the definition of *r*_high_ and *r*_low_.

Consider the five reflections shown schematically in figure 1*a*, in which the thickness of the excited region of reciprocal space has been exaggerated for clarity and to show the extreme situations of partiality even though they are not necessarily expected in real data. The filled circles A–E represent some hypothetical reflections which all have the same structure factor and hence would appear, when fully recorded under equivalent conditions, with the same intensity. Reflections B and C have very similar scattering angles, as do reflections D and E. The grey region represents the volume of reciprocal space within which reflections can be excited. Assuming that the X-ray beam is perfectly collimated and that its spectrum is a top hat function (i.e. all wavelengths within the bandwidth of the beam appear with equal intensity), reflections A and C would appear with approximately equal intensity, even though reflection A has a small partiality whereas reflection C is fully recorded. This can be seen by considering each of the Ewald spheres within the ‘nest’ individually: every one of them intersects both of the reflections, the only difference being that the central region of reflection A is excited, whereas the excited volume of reflection C includes the entire volume of the sphere. For the same reason, reflections B and E would also have similar intensities. Because the distance between the limiting Ewald spheres increases with scattering angle, the available incident intensity is spread over a larger volume of reciprocal space near the high-resolution reflections D and E than near the lower-resolution reflections B and C. Reflection D would therefore have a much lower intensity than reflection B, and E lower than C, despite both pairs of reflections having equal partiality. Reflections A, B and D all have similar partialities, yet they would appear with different intensities.

The diffracting extent for a particular reflection is defined by the distances *r*_high_ and *r*_low_, from which the partiality can be calculated. The definition of these distances is shown in figure 1*b*. They are the distances from the centre of the reciprocal lattice point to the limiting Ewald spheres, measured radially from the Ewald sphere centre. A negative value for either distance indicates that the surface of the corresponding limiting Ewald sphere is closer to the centre of the Ewald sphere than the reciprocal lattice point.

To account for the increasing distance between the limiting Ewald spheres with scattering angle, a ‘Lorentz’ factor was used in combination with the calculated partialities. The Lorentz factor is proportional to (*r*_high_ − *r*_low_)*^−^*^1^.

Before calculating the partialities, *r*_high_ and *r*_low_ were clamped to be within the range *−r …* +*r*, and this clamping taken into account for the later gradient calculation. This accounts for reflections such as reflection B in figure 1*a*, where moving the lower limiting Ewald sphere would not affect the partiality of the reflection, or reflection E where moving neither limiting Ewald sphere would affect the partiality.

The formula for converting the *e*th partially recorded intensity measurement in diffraction pattern *j*, *I_ej_*_,partial_ to a full intensity *I_ej_*_,full_ is therefore
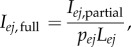
where *p* and *L* are the partiality and Lorentz factor, respectively. The cell parameters and orientations were represented together by using the *x*, *y* and *z* components of the reciprocal lattice basis vectors a*, b* and c*. The partiality itself is given by
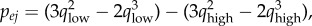
where 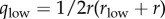, 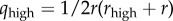 and *r* is the radius of the reflection sphere [8].

For the least-squares calculation at the core of post-refinement, the gradient of *p* with respect to the individual components of the basis vectors (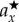, 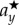, 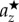, 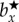 and so on) is required, and is given by
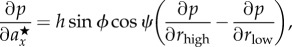

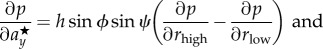

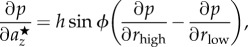
where
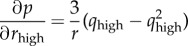
and
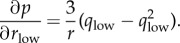


The expressions are similar for the components of b* and c* except that *k* and *l*, respectively, appear in the equation instead of *h*. The position of the reflection in the direction perpendicular to the nearest limiting Ewald sphere, which most strongly affects its partiality, does not strongly affect *L*. Therefore, the Lorentz gradient ∂*L*/∂*v*, where *v* represents any reciprocal lattice basis vector component, was taken to be zero so that 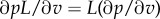.

## 3. Methods

**Figure 2.**
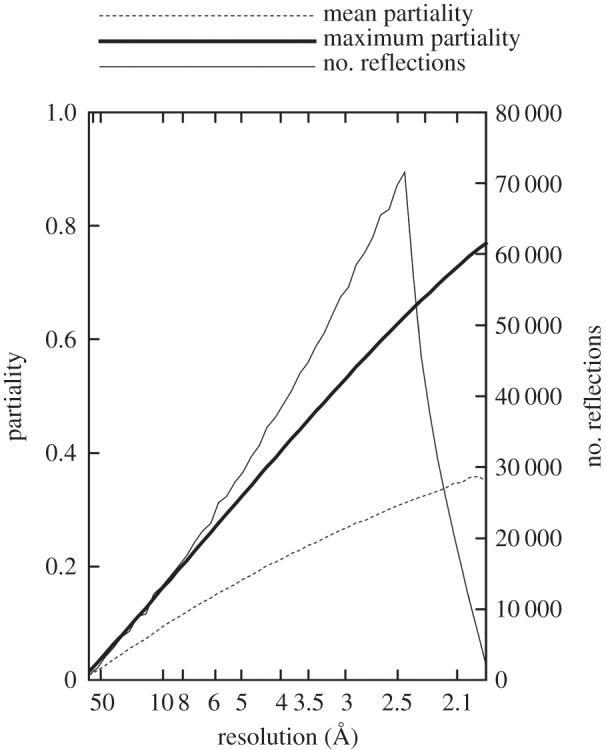
Mean and maximum partialities and number of reflections in the test dataset.

A simulated dataset was created by calculating partial intensities for 1000 randomly chosen crystal orientations using the model described above. Full intensities were calculated for PDB code 3PQR [9], which has a rhombohedral unit cell with *a* = 144.2 Å and *α* = 113.78°, space group R32, expressed using hexagonal axes (‘H32’). The simulated photon energy was 8 keV (wavelength 1.5498 Å), the bandwidth 0.05% and the convergence angle of the X-ray beam 1 mrad. The radius of the spheres of scattering density around each reciprocal lattice point was 5 × 10*^−^*^3^ nm*^−^*^1^. To simulate the limited accuracy of the initial geometrical parameters, errors were added to each of the reciprocal lattice basis vector components with a flat top distribution, maximum value ±0.1% of the component itself. The partial intensities were multiplied by an overall scaling factor, chosen randomly for each pattern according to a normal distribution with a mean of 1 and a standard deviation of 0.3. A square detector was simulated with side length 76.8 mm, a distance of 50 mm from the interaction point. Figure 2 shows the number of reflections, mean partiality and maximum partiality in resolution shells. The overall mean partiality was 0.25, and the maximum partiality of any reflection in the test dataset was 0.77. The largest number of reflections was encountered at the edge of the simulated detector where *d* = 2.41 Å, the steep fall-off at higher resolution corresponding to the corner regions. Normally distributed random noise was added to all reflections, with a constant standard deviation approximately equal to the mean intensity in the highest resolution shell. The resolution limit of the dataset was at *d* = 1.93 Å. A further dataset was prepared, identical to the first except for having a maximum reciprocal parameter error of 1.0% instead of 0.1%. Polarization of the X-ray beam was neglected in this simulation and its processing, but an additional correction factor could easily be used when handling experimental data.

To begin the scaling and post-refinement process, the fully integrated intensities for each symmetrically unique reflection (*I*_full_) were estimated by combining the many estimates from the *n* diffraction patterns using the parameters from the previous iteration
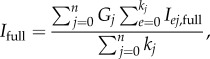
where the reflection has *k_j_* symmetry equivalents in image *j*, numbered *e* = 0 *… k_j_*, *G_j_* is the overall scaling factor for image *j*, and the expression for *I_ej_*_,full_ was given earlier. Reflections for which *p_ej_* < 0.1 were not included in the sum.

Before combining the intensity estimates, the overall scaling factors *G_j_* must be determined by a standard scaling procedure. The traditional matrix-based methods [10,11], which involve solving a square matrix equation with a side length equal to the number of patterns, are problematic when the number of patterns is very large, as is the case here, because the computational requirements scale in proportion to the square of the number of patterns. By contrast, the method described by Kabsch [12] operates by first merging all the patterns using equal scaling factors, then scaling each pattern to the combined dataset, merging the patterns again and iterating a small number of times. The computational requirements of this method are roughly proportional to the number of patterns, and it is therefore strongly preferred over the matrix-based method. A method similar to the Kabsch method was used in this work to scale and merge the individual diffraction patterns.

After combining the full intensity estimates from all patterns, the aim of post-refinement is to optimize the geometrical parameters such that the agreement is maximized between the partial intensity from a single reflection measurement, *I_ej_*_,partial_, and the corresponding fully integrated intensity, *I*_full_. The residual Δ*I_ej_* for a given partial reflection is therefore defined as
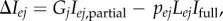
where *p_ej_* is the partiality of the reflection calculated using current estimates of the diffraction parameters. The object is to minimize the sum of the squared residuals from all reflections in the pattern3.1
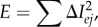
which was performed by nonlinear least-squares fitting. Any reflection was omitted from the least-squares procedure whose intensity was less than three times the estimated error in the intensity, if its partiality *p_ej_* < 0.1, or if there was not at least one other scalable measurement of the reflection in the dataset (whether in the same pattern or not). If no reflections remained in particular pattern after these rejection criteria had been applied, then no refinement was performed on that pattern. The nine reciprocal lattice basis vector components were used as parameters for the refinement. The normal equations were solved using singular value decomposition (SVD), after applying the rescaling procedure described by Bricogne [13], which ensures that the diagonal elements of the matrix are all 1, and therefore that any small eigenvalues were due only to correlations between parameters and not owing to differences of scale. Eigenvalues were eliminated if their moduli were less than 10*^−^*^6^ times the largest eigenvalue. Iteration was continued until the largest change in partiality was less than 0.01, or to a maximum of 10 iterations, whichever came first. If the step taken by an iteration caused more than one third of the reflections to be assigned partialities of zero (i.e. determined not to appear in the pattern), the step was reverted. The procedure was then repeated from the scaling and merging step, using the refined geometrical parameters to calculate new partialities and Lorentz factors.

The procedure was tested with zero, one and three cycles of the procedure, where zero iterations correspond so performing the initial scaling and merging step alone, using the initial reciprocal lattice basis vectors to estimate partialities, and skipping the post-refinement step altogether. For each trial of the procedure, the accuracy of the resulting estimates of the fully integrated intensities was evaluated by calculating the *R*-factor in resolution shells between them, and the ‘reference intensities which were used to create the test data at the beginning’.

## 4. Results

**Figure 3.**
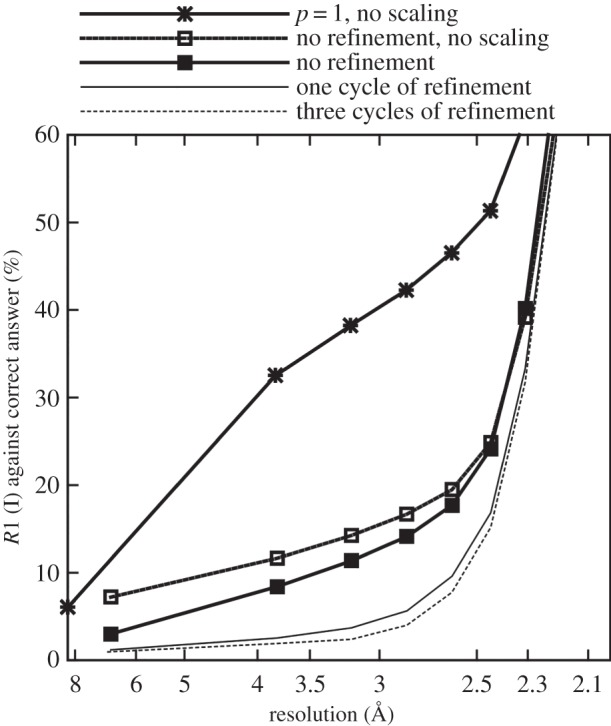
Comparison of *R*-factors with a maximum initial reciprocal space error of 0.1%.

Figure 3 shows the results of the application of the scaling and post-refinement procedure to the first simulated test dataset (maximum reciprocal parameter error ±0.1%). Three cycles of the post-refinement procedure give the best agreement between the reference intensities and the merged intensities, with one cycle of the procedure performing only slightly less well. Post-refinement made an improvement to the data quality in all resolution shells up to the resolution limit of the data.

The results with *p* = 1 (i.e. with all partialities set to 1) extend to slightly lower resolution than the others because of the rejection of reflections with very small partialities, which are more frequently found at the very lowest resolutions. One eigenvalue was found to have been eliminated during each iteration of the filtering procedure after SVD, consistent with the finding of Rossmann *et al.* [8] that a rotation of the crystal around the direction of the incident X-ray beam does not affect the partialities and so leads to a redundancy in the refinable parameters. Although the least-squares problem was expressed using the Cartesian components of the basis vectors in this work, there is a linear relationship between these and the setting angles and cell parameter representation used in the earlier work, and so the same problem occurs. The least-squares fitting procedure was nevertheless able to continue and refine the parameters.

**Figure 4.**
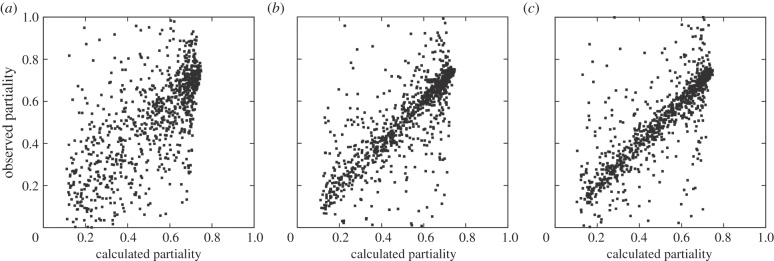
Correlation between observed and calculated partialities after (*a*) zero, (*b*) one and (*c*) three cycles of post-refinement.

For a given reflection, the ‘observed partiality’ can be defined as the ratio of the intensity in the pattern, after correction for the overall scaling and Lorentz factors (but not partiality), to the current estimate of the full intensity. These values were plotted in a scatter graph against the ‘calculated partiality’, which is the estimate *p_ej_* from the geometrical model, shown in figure 4. To reduce the number of points in the graphs for clarity, points were randomly selected from the entire dataset with a probability chosen to include approximately the number of reflections found in a single diffraction pattern. The graphs show that a weak correlation is visible before refinement, and that the correlation becomes much stronger after one iteration.

**Figure 5.**
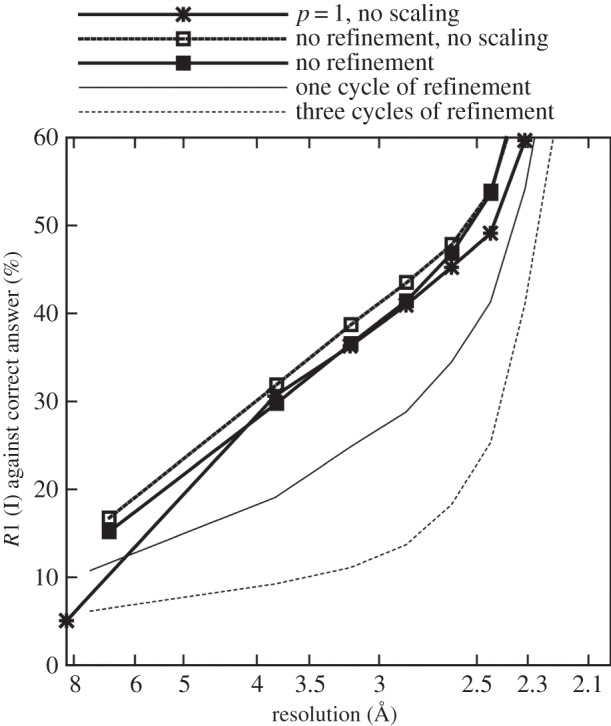
Comparison of *R*-factors with a maximum initial reciprocal space error of 1.0%.

The results shown in figure 3 with ‘no refinement’ are significantly better than those with *p* = 1, indicating that under these conditions the parameters were still sufficiently accurate that they could be used to make useful partiality estimates without post-refinement. The gap between the ‘no refinement, no scaling’ and ‘no refinement’ lines indicates the amount of error introduced by the distribution of overall scaling factors under these conditions. For the second test dataset (maximum reciprocal parameter error of ±1.0%), the initial parameters were not accurate enough to produce useful partiality estimates, as can be seen in figure 5. This is a much more challenging situation for the post-refinement algorithm, but it was still able to make a significant improvement to the data quality.

## 5. Conclusion

It has been shown that a post-refinement procedure can be used to improve the parameters of a geometrical model of partiality when the entire dataset consists of partially recorded reflections. The refinement can be performed in a stable manner, and works even when the initial estimates of the parameters are very far from the true values. The refined parameters can be used to assign partialities to the reflections which allow significant improvements in the data quality compared with when no partiality estimates are made.

This demonstration used simulated data, and the partiality model used during post-refinement was the same as that used to produce the test data at the beginning. Future work on this subject will consist of tests with diffraction patterns simulated using Fourier methods [3], indexed and integrated using the established SFX analysis pipeline, and on experimental data from SFX experiments. Applying this method to experimental data is likely to require first achieving a greater understanding of the importance of the spectral fluctuations arising from the stochastic mode of FEL operation, as well as accounting for vast differences in diffraction strength and resolution between crystals, and perhaps several additional confounding factors. Nevertheless, these simulations still serve to demonstrate the above points concerning the practicality and stability of the method, which is likely to be of great importance for improving the data quality which can be achieved using a given number of patterns in an SFX experiment.

The post-refinement method has been implemented as part of the serial femtosecond crystallography suite CrystFEL [14], embodied in the program *partialator.* The generation of test data was performed using the CrystFEL program *partial_sim*. The results described in this article may be reproduced by using version 0.5.2 of CrystFEL.

## References

[1] Chapman HN 2011 Femtosecond X-ray protein nanocrystallography. Nature 470, 73–77. (10.1038/nature09750) 21293373 PMC3429598

[2] Neutze R Wouts R van der Spoel D Weckert E Hajdu J 2000 Potential for biomolecular imaging with femtosecond X-ray pulses. Nature 406, 752–757. (10.1038/35021099) 10963603

[3] Kirian RA 2010 Femtosecond X-ray protein nanocrystallography: data analysis methods. Opt. Express. 18, 5713–5723. (10.1364/OE.18.005713) 20389587 PMC4038330

[4] Kirian RA 2011 Structure-factor analysis of femtosecond micro-diffraction patterns from protein nanocrystals. Acta Crystallogr. A 67, 131–140. (10.1107/S0108767310050981) 21325716 PMC3066792

[5] Rossmann MG van Beek CG 1999 Data processing. Acta Crystallogr. D 55, 1631–1640. (10.1107/S0907444999008379) 10531511

[6] Winker FK Schutt CE Harrison SC 1979 The oscillation method for crystals with very large unit cells. Acta Crystallogr. A 35, 901–911. (10.1107/S0567739479002035)

[7] White TA Barty A Stellato F Holton JM Kirian RA Zatsepin NA Chapman HN 2013 Crystallographic data processing for free electron laser sources. Acta Crystallogr. D 69, 1231–1240. (10.1107/S0907444913013620) 23793149 PMC3689526

[8] Rossmann MG Leslie AGW Abdel-Meguid SS Tsukihara T 1979 Processing and postrefinement of oscillation camera data. J. Appl. Crystallogr. 12, 570–581. (10.1107/S0021889879013273)

[9] Choe HW Kim YJ Park JH Morizumi T Pai EF Krauss N Hofmann KP Scheerer P Ernst OP 2001 Crystal structure of metarhodopsin II. Nature 471, 651–655. (10.1038/nature09789) 21389988

[10] Hamilton WC Rollett JS Sparks RA 1965 On the relative scaling of X-ray photographs. Acta Crystallogr. 18, 129–130. (10.1107/S0365110X65000233)

[11] Fox GC Holmes KC 1966 An alternative method of solving the layer scaling equations of Hamilton, Rollett and Sparks. Acta Crystallogr. 20, 886–891. (10.1107/S0365110X66002007)

[12] Kabsch W 2010 Integration, scaling, space-group assignment and post-refinement. Acta Crystallogr. D 66, 133–144. (10.1107/S0907444909047374) 20124693 PMC2815666

[13] Bricogne G 1986 The importance of rescaling the normal matrix before eigenvalue filtering. In Proc. the EEC Cooperative Workshop on Position-Sensitive Detector Software (Phase III), Orsay, November 1986 pp. 65–68. LURE.

[14] White TA Kirian RA Martin AV Aquila A Nass K Barty A Chapman HN 2012 CrystFEL: a software suite for snapshot serial crystallography. J. Appl. Crystallogr. 45, 335–341. (10.1107/S0021889812002312)

